# Precise Fabrication of Porous Microspheres by Iso-Density Emulsion Combined with Microfluidics

**DOI:** 10.3390/polym14132687

**Published:** 2022-06-30

**Authors:** Yuxiao Shi, Xin Zhang, Ketao Mu, Yifan Wang, Ting Jiang, Shangtong Jiang, Shengmin Zhang, Yingying Du

**Affiliations:** 1 Advanced Biomaterials and Tissue Engineering Center, Huazhong University of Science and Technology, Wuhan 430074, China; shiyxcn@hust.edu.cn (Y.S.); zhangxin5@hust.edu.cn (X.Z.); aquariusoul@126.com (Y.W.); jiangting1999@hust.edu.cn (T.J.); m202071931@hust.edu.cn (S.J.); smzhang@hust.edu.cn (S.Z.); 2 NMPA Research Base of Regulatory Science for Medical Devices & Institute of Regulatory Science for Medical Devices, Huazhong University of Science and Technology, Wuhan 430074, China; 3 Department of Biomedical Engineering, Huazhong University of Science and Technology, Wuhan 430074, China; 4 Department of Radiology, Tongji Hospital, Tongji Medical College, Huazhong University of Science and Technology, Wuhan 430030, China; muketao@163.com; 5 School of Stomatology, Tongji Medical College, Huazhong University of Science and Technology, Wuhan 430030, China

**Keywords:** porous microspheres, stable emulsion, double emulsion template, microfluidics, polycaprolactone

## Abstract

Polymer porous microspheres with large specific surface areas and good fluidity have promising important applications in the biomedical field. However, controllable fabrication of porous microspheres with precise size, morphology, and pore structure is still a challenge, and phase separation caused by the instability of the emulsion is the main factor affecting the precise preparation of porous microspheres. Herein, a method combining the iso-density emulsion (IDE) template and microfluidics was proposed to realize the controllable preparation of polymer porous microspheres. The IDE exhibited excellent stability with minimal phase separation within 4 h, thus showing potential advantages in the large-scale preparation of porous microspheres. With the IDE template combined microfluidics technique and the use of a customized amphoteric copolymer, PEG-b-polycaprolactone, polycaprolactone (PCL) porous microspheres with porosity higher than 90% were successfully prepared. Afterwards, the main factors, including polymer concentration, water–oil ratio and homogenization time were investigated to regulate the pore structure of microspheres, and microspheres with different pore sizes (1–30 μm) were obtained. PCL porous microspheres exhibited comparable cell viability relative to the control group and good potential as cell microcarriers after surface modification with polydopamine. The modified PCL porous microspheres implanted subcutaneously in rats underwent rapid in vivo degradation and tissue ingrowth. Overall, this study demonstrated an efficient strategy for the precise preparation of porous microspheres and investigated the potential of the as-prepared PCL porous microspheres as cell microcarriers and micro-scaffolds.

## 1. Introduction

With high specific surface area and good fluidity, polymer porous microspheres have played an important role in a wide range of biomedical applications, such as controllable drug delivery, adsorbent, cell delivery, and enzyme immobilization [1,2,3,4,5,6]. The employment of microfluidics has facilitated the development of various solid microspheres and particles due to the enhanced precision and controllability [7,8,9,10,11]. However, precise fabrication of porous microspheres is still a challenge since the rapid phase separation damages the emulsion template and further impairs the uniformity of the microspheres in particle size and pore structure [12], which limits the yield and application of porous microspheres. Therefore, the stability of the emulsion template is crucial for the preparation of porous microspheres. Although emulsion stabilized by emulsifiers, solid particles, as well as amphiphilic block copolymers have been widely reported [13,14,15,16,17], most of them are not suitable for the fabrication of porous microspheres due to the introduction of additional compositions. One important factor driving the phase separation is drainage [18], which will be aggravated with the increase of density difference between the water phase and oil phase in emulsion. Therefore, this study proposed a density regulation method for emulsion stabilization, which will be a potentially effective strategy for the controllable preparation of porous microspheres.

In addition to the stability of emulsion, properties of the polymers, such as hydrophobicity and mechanical properties, are also crucial for pore formation. It has been widely reported that poly(D, L-lactide-co-glycolide) (PLGA) and polylactic acid (PLA) porous microspheres could be obtained without surfactants [4,5,19,20]. However, hydrophobic polymers, such as polycaprolactone (PCL), are unlikely to form connected pores [21,22]. Partial hydrolysis of polymers has been shown to provide hydrophilic groups and promote the formation of surface pores [21,23], while amphoteric polymers are more effective to adjust the surface morphology of the microspheres [24,25,26]. Nonetheless, amphiphilic copolymers containing PEG blocks have been reported to resist cell adhesion and protein adsorption since the highly hydrated PEG chains could form an antifouling layer [27]. To solve this problem, binding adhesion peptides and polydopamine (PDA) surface modification were reported to promote cell adhesion [28,29,30,31,32,33].

**Figure 1:**
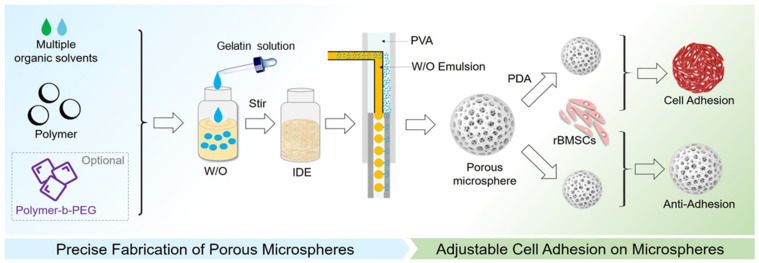
Schematic illustration of the preparation of iso-density emulsion (IDE), the fabrication of porous microsphere, and PDA modification for cell adhesion.

Herein, an improved method for porous microsphere preparation that combined iso-density emulsion (IDE) and microfluidics was demonstrated (Figure 1). Two kinds of organic solvents were used to reduce the density difference between water phase and oil phase as to prevent the phase separation of emulsion. Firstly, an emulsion stability test was conducted to show the minimal phase separation over time in iso-density emulsion. Then, the porous microspheres derived from IDE exhibited excellent uniformity. Furthermore, PCL porous microspheres were prepared with the addition of MePEG-b-PCL (PECL), and the effects of the main factors on the morphology of microspheres were investigated to achieve the precise control of the microsphere structure. In addition, we evaluated the cytocompatibility of PCL microspheres and PDA-modified PCL microspheres. Finally, PDA-modified PCL porous microspheres were implanted subcutaneously into rats by minimally invasive injections for the investigation of histocompatibility and degradation in vivo.

## 2. Materials and Methods

### 2.1. Materials

Poly(lactic-co-glycolic acid) (PLGA) ([η] = 0.62 dL/g) and monomethoxy-poly(ethylene glycol)-b-polycaprolactone (MePEG-b-PCL, PECL) (*M*_w_ = 45,000) with a MePEG block of 2000 in molecular weight were both purchased from Shandong Academy of Pharmaceutical Sciences (China). Polycaprolactone (PCL) (*M*_w_ = 45,000; Aldrich), polyvinyl alcohol (PVA) (*M*_w_ = 30,000–70,000, 87–90% hydrolyzed; Sigma-Aldrich), and dopamine hydrochloride (Aladdin) were commercially available. Dichloromethane (DCM), gelatin, and Span 80 were all purchased from Sinopharm Chemical ReagentCo., Ltd. (Shanghai, China).

### 2.2. Emulsion Stability Test

The emulsion stability test was carried out to show the stability of emulsions, according to the published work [12]. Phase separation of iso-density emulsion (IDE) and normal emulsion without (NE1) or with (NE2) Span 80 over time were observed, respectively, to test the stability of the emulsion. For IDE, PCL (2.5%, *w*/*w*) in dichloromethane (DCM) and ethyl acetate (EA) (5/8, *w*/*w*) served as the oil phase (O), while the aqueous solution of gelatin (7.5%, *w*/*w*) served as the water phase (W). For NE1 and NE2, only DCM was employed as the organic solvents to dissolve PCL without or with 3% Span 80. All emulsions (8 mL) were prepared via magnetic stirring at 2200 rpm for 3 min followed by emulsification using a homogenizer (FJ200, Shanghai Biaoben Mould Factory, China) at minimum speed for 24 s, then placed under 25 °C and photographed at a series of time points for statistics (*n* = 3). The stability of the emulsion is quantified by measuring the quality of the emulsion over time, calculated according to the following formula:

Quality of emulsion = height of emulsion layer/total height × 100%(1)

### 2.3. Preparation of Uniform Porous Microspheres

According to the published works [3], the microfluidic device for porous microsphere preparation consists of two pumps, two glass syringes, a right-angled stainless-steel needle, silicone tubes, and a customized glass capillary. Herein, the needle was just inserted into one end of the capillary in a silicone tube, while another end of the capillary was submerged in the iced collection phase. W/O emulsion was introduced as the discontinuous phase into the microfluidic device, then the discontinuous phase from the needle, and the continuous phase (2% PVA) from the silicone tube were converged at the capillary at an appropriate flow rate to generate W/O/W droplets at the tip of the needle. The droplets flowed along the capillary into the iced collection phase and were stirred gently, where the gelatin solution in droplets became gel as porogen, and the organic solvents in the droplets dissipated into the collection phase and evaporated, which resulted in the formation of microspheres with gelatin embedded in it. After sufficient evaporation, the microspheres were collected and immersed into 45 °C distilled water under gentle stirring at 100 rpm for 1 h to remove the gelatin porogen completely. Finally, porous microspheres were washed with distilled water repeatedly to remove residual PVA and then were freeze-dried for 24 h.

Herein, PLGA porous microspheres were prepared for the uniformity test since the regular pores on the surface facilitate the statistical analysis of pore size distribution. For IDE, PLGA (3 wt%) in DCM&EA (5/8, *w*/*w*) served as O and the aqueous solution of gelatin (7.5 wt%) served as W. W/O emulsion (W/O = 1/2.4, *w*/*w*) was prepared via preliminary emulsification with a magnetic stirrer and then homogenization for 24 s with a high-speed homogenizer. As for NE, only DCM was used in O. IDE and NE were then introduced into the microfluidic device, respectively, in which a 26 G needle and glass capillary with inner diameter of 0.5 mm were employed. Afterwards, PLGA porous microsphere preparation was conducted, as mentioned above. The obtained microspheres were dispersed in water and photographed under an optical microscope for statistics on particle size distribution, while the surface morphologies of the freeze-dried microspheres were observed under a field-emission SEM (FE-SEM, S-4800, HITACHI, Tokyo, Japan) for statistics on pore size distribution. A total of 100 microspheres were randomly selected from three kinds of microspheres for pore size distribution statistics.

### 2.4. Preparation of PCL Porous Microspheres with Controllable Morphology

With an amphoteric polymer PECL serving as the surfactant, the PCL porous microspheres could be prepared. The effects of the main fabrication parameters, including PCL and PECL concentrations in O (C_PCL_ and C_PECL_), W/O ratio (R_W/O_), homogenization time (T_H_) as well as capillary inner diameter (ID) on morphology of porous microspheres were investigated, respectively, for obtaining precise controllable porous microspheres. All obtained microspheres were observed under a field-emission SEM. 

### 2.5. Characterization of PCL Porous Microspheres

The porosity measurement, XRD, FTIR, TGA, and DSC test of PCL porous microspheres (Table 1, microspheres g) in which the pure PCL and PECL served as the controls were conducted. The in vitro degradation test of the PCL porous microspheres was also carried out. The density of the PCL porous microspheres was measured via the mass–volume method with removal of space between microspheres for porosity measurement using the following formula:

Porosity = 6/π × (Mass of microspheres)/(Volume of microspheres) × 1/(Density of PCL) × 100%(2)

XRD analysis ranging from 5 to 50° was carried out using x’pert3 powder (PANalytical B.V., Almelo, Netherlands) at 40 kV and 40 mA in which the radiation source was Cu Kα, λ = 1.54060 Å, and the scan step was 0.01313°. FTIR analysis ranging from 4000 to 400 cm^−1^ was carried out using Nicolet iS50R (Thermo Scientific, Waltham, MA, USA) in which the resolution was 0.482 cm^−1^. The TGA ranging from room temperature to 500 °C was carried out using Pyris1 TGA (PerkinElmer Instruments, Waltham, MA, USA) at a heating rate of 10 °C/min in a nitrogen atmosphere. Additionally, the melting temperature, enthalpy of fusion (Δ*H_m_*), and degree of crystallinity (*xc*) were characterized by the DSC test, using Diamond DSC (PerkinElmer Instruments, Waltham, MA, USA). A constant nitrogen flow was induced to increase the temperature from 30 °C to 150 °C, followed by cooling down to 30 °C and reheating to 150 °C in which the temperature change rate was 10 °C/min. The results from the second heating were adopted to eliminate the thermal history [34]. The degree of crystallinity could be obtained by the following formula [35]:

xc = (Δ*H_m_* of sample)/(Δ*H_m_* of 100% crystalline PCL) × 100% (3)

where the Δ*H_m_* of 100% crystalline PCL was taken at 136.5 J g^−1^ [36].

At last, the in vitro degradation test of the PCL porous microspheres was carried out via putting 0.02 g freeze-dried microspheres in 8 mL PBS and then placing in a shaker with 200 rpm at 37 °C. The weight of the microspheres and the pH of the degradation solution were measured every week.

### 2.6. Cytotoxicity Test of PCL Porous Microspheres

Mesenchymal stem cells derived from rat bone (rBMSCs, purchased from Cyagen Biosciences Inc., USA) were used to evaluate the cell response to porous microspheres. rBMSCs were cultured in Dulbecco’s modified Eagle medium (DMEM) supplemented with 10% (*v*/*v*) fetal bovine serum and 1% (*v*/*v*) penicillin/streptomycin antibiotics (all purchased from Gibco). To investigate the cytotoxicity of the PCL porous microspheres containing PECL, 5000 cells were seeded and co-cultured with 100 porous microspheres in a 96-well plate, while cells co-cultured with PCL solid particles and cells alone served as the control. After incubation for predetermined times (3, 5 and 7 d), the medium containing microspheres was removed, and the cells were washed with warm PBS. Finally, the cells were observed under an optical microscope. Meanwhile, the 100 μL medium supplemented with 10% (*v*/*v*) CCK-8 working solution was added into each well and incubated at 37 °C for 1 h. The optical density (OD) value was obtained by using a microplate reader at 450 nm.

### 2.7. Dopamine Surface Modification and Cell Adhesion of PCL Porous Microspheres

The cell adhesion test of PCL porous microspheres with or without PDA modification was carried out to reveal the antifouling ability of PECL in microspheres and the cell adhesion ability after PDA modification. The PCL porous microspheres (Table 1, microspheres g) were modified via PDA and then co-cultured with rBMSCs to investigate the cell adhesion, while PDA-free PCL porous microspheres (PMs) served as the control. Firstly, microspheres were dispersed in 10 times the volume of Tris buffer (0.25 mM, pH = 8.5) supplemented with 0.5 mg/mL dopamine hydrochloride and incubated in a shaker (100 rpm) at 37 °C for 12 h to obtain PDA modified PCL porous microspheres (PDPMs). Afterwards, PDPMs were sterilized by UV and dispersed in 20 times the volume of medium to prepare 10% *v*/*v* suspension. Finally, 400 μL suspension of PDPMs was added into each well of the 48-well plate (*n* = 4) and rBMSCs were seeded with a density of 40,000 cells per well. Cell adhesion on PMs was also conducted as a control. After incubation for the predetermined times (1, 3, 5 and 7 d), PDPMs and PMs were collected, washed twice with PBS, fixed in 4% paraformaldehyde for 15 min, and stained with Rhodamine Phalloidin as well as DAPI, according to the manufacturer’s instructions. After staining, PDPMs and PMs were observed under CLSM (FV3000).

### 2.8. In Vivo Compatibility and Degradation of PCL Porous Microspheres

All procedures for animal experiments were approved by the Institutional Animal Care and Use Committee (IACUC) of Huazhong University of Science and Technology. SD rats (weighing 100–110 g, four weeks old) were used in the animal experiment. PDA-modified PCL porous microspheres were suspended in saline at a volume ratio of 1/4 and implanted subcutaneously in rats immediately via minimally invasive injections, followed by penicillin injections. The implants were harvested after one, three, and five weeks for paraffin-embedded sectioning and H&E staining, then the compatibility and degradation of the implants were observed. The animal experiment was approved by the Institutional Animal Care and Use Committee (IACUC) of Huazhong University of Science and Technology.

## 3. Results and Discussion

### 3.1. Emulsion Stability

**Figure 2:**
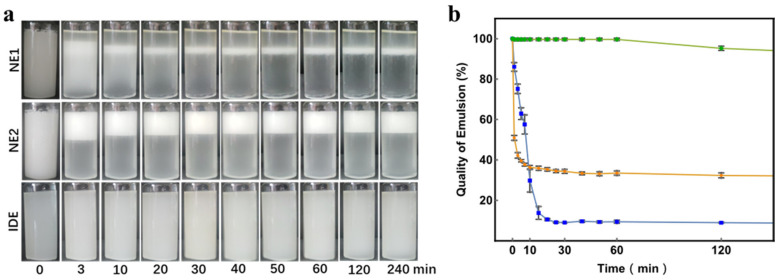
(**a**) Phase separation of NE1, NE2, and IDE at different time points; (**b**) corresponding emulsion quality of NE1, NE2, and IDE over time (*n* = 3).

The photos of the emulsions and the statistics results (*n* = 3) of the quality of emulsion over time are shown in Figure 2. As anticipated, normal emulsion without Span 80 (NE1) stratified rapidly, resulting in a water-rich layer on the top, a W/O emulsion layer in the middle, and an oil-rich layer at the bottom, while the quality of NE1 dramatically dropped to 8.3%. Despite the use of Span 80 in NE2 resulting in a slightly improved emulsion quality of 31.5%, stratification failed to be waived. The instability of NE1 and NE2 could be mainly attributed to the great difference in the density between the oil and water phase. On the contrary, the iso-density emulsion (IDE) showed excellent stability without visible phase separation even after 4 h of placement, and the emulsion quality maintained above 90%. The phase separation in IDE was not significant since the minimal difference in the density between the oil and water phase would be counteracted by interface force. Therefore, IDE has advantages in large-scale preparation of porous microspheres.

### 3.2. Uniformity of Porous Microspheres

**Figure 3:**
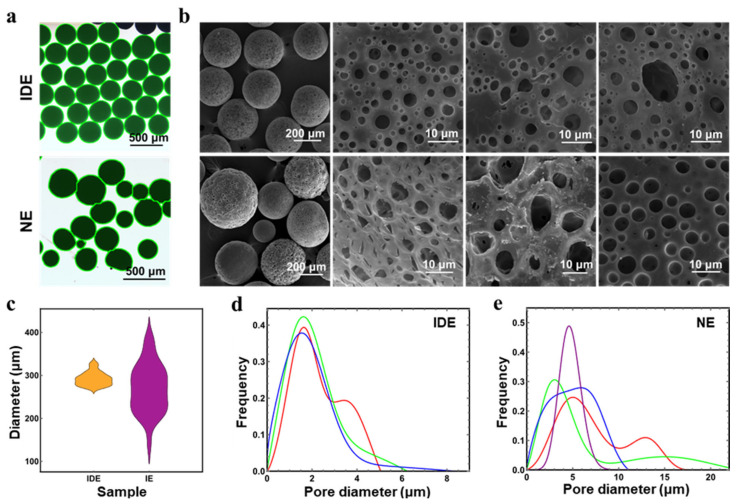
(**a**) Optical microscope photos of microspheres derived from IDE and NE; (**b**) SEM images of microspheres; (**c**) particle size distribution of the microspheres; (**d**,**e**) pore size distributions of microspheres derived from IDE and NE.

PLGA porous microspheres were used to test the uniformity of microspheres. The optical microscope photos (microspheres identified by software and employed in statistics are shown in green highlight) and the SEM images of PLGA porous microspheres derived from IDE and NE are shown in Figure 3a,b, while particle size distribution and pore size distribution are shown in Figure 3c,e. Obviously, particle size distribution and pore size distribution of microspheres derived from IDE were concentrated, which indicated the enduring stability of IDE with minimal phase separation. By contrast, the microspheres derived from NE had a wide distribution in particle size and pore size, and visible phase separation was observed during microspheres preparation. Therefore, IDE has great ad-vantages in the preparation of porous microspheres with uniform size and morphology.

### 3.3. PCL Porous Microspheres with Controllable Morphology

**Figure 4:**
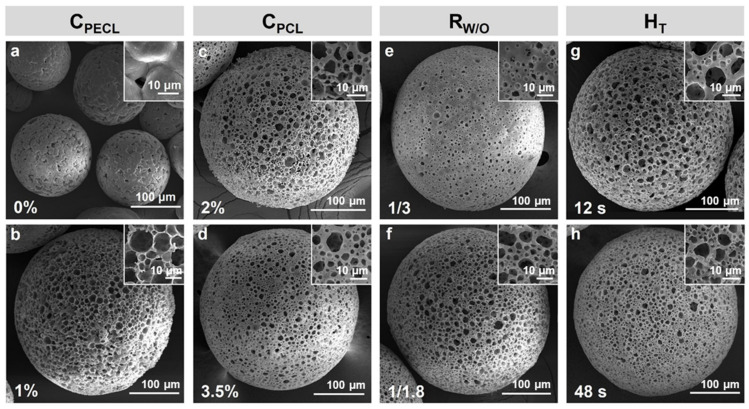
Microspheres prepared with different parameters. (**a**,**b**) with C_PECL_ of 0% and 1%; (**c**,**d**) with C_PCL_ of 2% and 3.5%; (**e**,**f**) with R_W/O_ of 1/3 and 1/1.8; (**g**,**h**) with H_T_ of 12 s and 48 s. C_PECL_ (PECL concentration, *w*/*w*), C_PCL_ (PCL concentration, *w*/*w*), R_W/O_ (mass ratio of W to O), and T_H_ (homogenization time).

**Figure 5:**
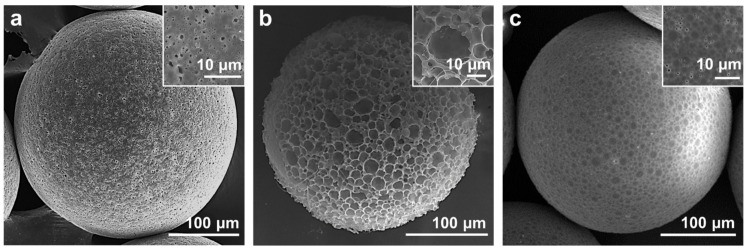
(**a**–**c**) Typical microspheres with different pore size and morphology.

The parameters of different PCL porous microspheres are shown in Table 1, and their SEM images are shown in Figure 4, correspondingly. Microspheres a-b, c-d, e-f, and g-h are microspheres with low or high C_PECL_ (PECL concentration, *w*/*w*), C_PCL_ (PCL concentration, *w*/*w*), R_W/O_ (mass ratio of W to O), and T_H_ (homogenization time), respectively. Customized microspheres with different pore size and morphology are shown in Figure 5.

Among the factors, C_PECL_ was found to affect the morphology of microspheres most significantly. Without PECL (Table 1, microsphere a), it was observed under an optical microscope that gelatin micelles escaped explosively from W/O droplets due to the hydrophobicity of PCL, producing microspheres that failed to form connected pores. Instead, irregular gaps and small depressions were observed (Figure 4a). With PECL, C_PECL_ of 1% (Table 1, microsphere b) would stabilize the emulsion template effectively since the hydrophilicity of PEG blocks in PECL could imprison the gelatin micelles, producing a large number of rounded pores (Figure 4b). Therefore, PECL could improve porosity and pore shape as a surfactant, but it would also reduce pore connectivity at a high concentration as a polymer.

Then, C_PCL_ was also found to be associated with the formation of microsphere morphology. When a low C_PCL_ of 2% (Table 1, microsphere c) was employed, the pores became slightly larger and the surface of microspheres became rougher (Figure 4c). With C_PCL_ increasing to 3.5% (Table 1, microsphere d), a trend of pores becoming small and the surface becoming smooth was observed (Figure 4d), which could be attributed to the thickening of pore walls. Therefore, C_PCL_ could be used to control the opening or closing of pores.

Afterwards, R_W/O_ showed significant effects on porosity and morphology of microspheres. With a low R_W/O_ of 1/3 (Table 1, microsphere e), the pores were observed to be smaller and sparse with poor connectivity (Figure 4e). With R_W_ increasing to 1/1.8 (Table 1, microsphere f), the pores became larger, denser, and more connected (Figure 4f), which could be attributed to the increase of gelatin as the porogen. Therefore, R_W/O_ could be used to control the porosity of microspheres.

Finally, the effects of T_H_ on pore diameter were also investigated. With a short T_H_ of 12 s (Table 1, microsphere g), pores were observed to be larger and rounded with great connectivity (Figure 4g). With T_H_ increasing to 48 s (Table 1, microsphere h), a limited reduction in pore diameter was observed (Figure 4h), which could be attributed to the automatic combination of gelatin in the presence of limited surfactant PECL. Overall, T_H_ alone could be used to control the pore diameter within a certain range.

Overall, the preparation of PCL porous microspheres with controllable morphology could be accomplished, and the size of porous microspheres could be simply controlled by using the microfluidic device of specific size.

### 3.4. Characterization of PCL Porous Microspheres

**Figure 6:**
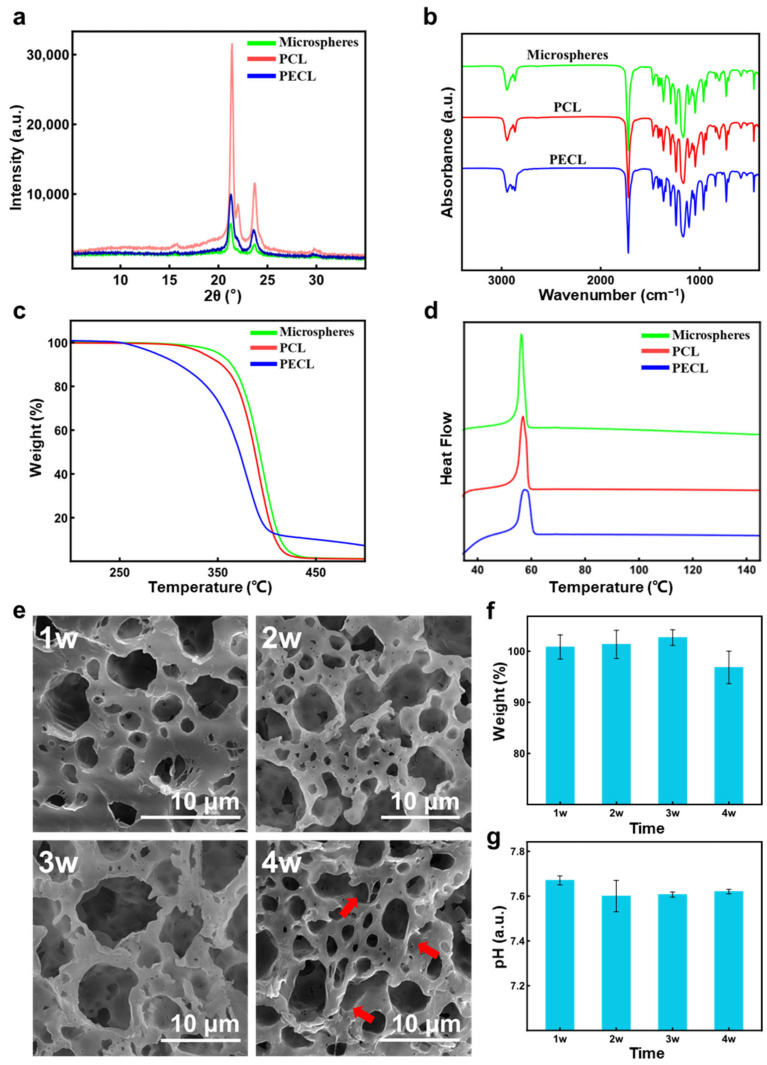
(**a**–**d**) The results of XRD, FTIR, TGA, and DSC test of PCL porous microspheres, PCL and PECL; (**e**–**g**) the changes of morphology (**e**) and weight of microspheres (**f**) as well as pH changes of degradation liquid (**g**) during the in vitro degradation.

The mass of 1 mL PCL porous microspheres was measured to be 0.0512 g, and the density of PCL was about 1 g/mL; it could be calculated that porosity of the PCL porous microspheres is about 90.22%, according to the formula 2. Then, XRD results (Figure 6a) showed that PCL had a high narrow diffraction peak at 21°, and there was a lower diffraction peak at 24°. It was a typical XRD result of PCL, which was similar to the results from other researches [37,38]. PECL was also observed to have these two diffraction peaks, but the strength is significantly reduced, indicating that the presence of the PEG block was not conducive to crystallization. The peak strength of the porous microspheres was the lowest, which could be attributed to the mixing of two polymers, further hindering the crystallization, and the plasticization of organic solvents during the preparation would also tend to make the material amorphous. Next, the FTIR results (Figure 6b) showed that the infrared absorption spectrograms of porous microspheres, PCL and PECL were similar. The characteristic bands of PCL were observed: the peaks at about 2950 and 2850 cm^−1^ indicated asymmetric CH_2_ and symmetric CH_2_; the strong peak at about 1700 cm^−1^ indicated the C=O; and the multiple peaks in the 1000–1250 cm^−1^ area could be attributed to the vibration of C-O and C-C [38]. Therefore, the preparation process of porous microspheres had no significant effects on the chemical composition. Afterwards, the TGA results (Figure 6c) showed that porous microspheres had similar thermal decomposition curves to PCL, while PECL began to decompose at a lower temperature, which could be attributed to the presence of the PEG block. DSC thermograms (Figure 6d) of porous microspheres showed a single endothermic peak between 56–58 °C, indicating the miscibility of PCL and PECL [39]. Since the presence of PECL was not conducive to the crystallization of PCL, decrease in the degree of crystallinity (47.52 to 43.26%) and Δ*Hm* (64.86 J g−1 to 59.05 J g−1) was observed. Lastly, the results of the in vitro degradation (Figure 6e–g) showed that the weight loss of PCL porous microspheres began at four weeks when the dissolution of the microsphere surface (marked with red arrows) was also observed under SEM. Compared with PLGA and PLA porous microspheres in other research [4,40], PCL porous microspheres had a slower degradation, but it was much faster than the degradation of solid PCL. The pH of the degradation solution did not change significantly over time, which indicated that PCL porous microspheres would not produce acidic degradation products, different from the porous microspheres made of PLGA and PLA.

### 3.5. Cytotoxicity Evaluation

**Figure 7:**
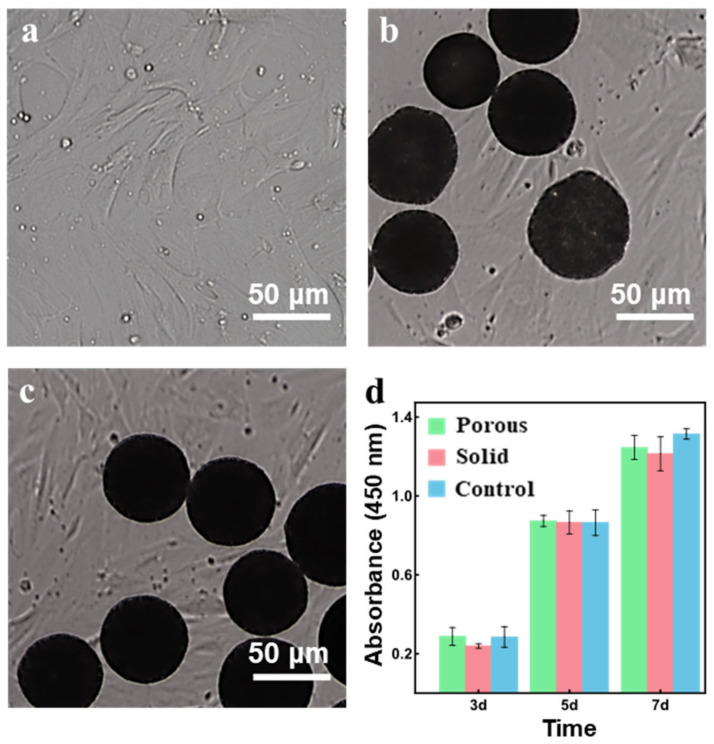
(**a**) rBMSCs alone and co-cultured with solid (**b**) or porous (**c**) microspheres at 7d; (**d**) cell activity and proliferation of rBMSCs during the co-culture.

The results of the CCK-8 test were shown in Figure 7. After three, five, and seven days of co-culture with rBMSCs, the porous microsphere group (Porous) exhibited consistent cell proliferation trends compared with the solid microsphere group (Solid) and rBMSCs alone (Control). No significant differences were observed among the three groups, indicating the porous PCL microspheres could well support cell proliferation. 

### 3.6. Polydopamine Modification and Cell Adhesion

**Figure 8:**
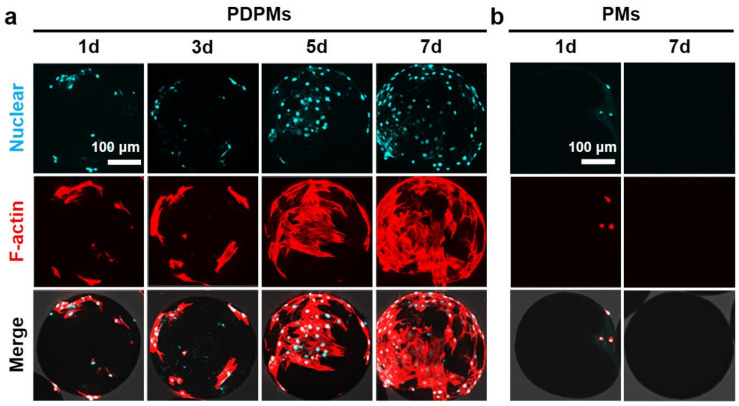
(**a**) Cell adhesion on PDA-modified PCL porous microspheres (PDPMs); (**b**) cell adhesion on PDA-free microspheres (PMs). (Scale bar is for all results in (**a**,**b**)).

The results of the cell adhesion test are shown in Figure 8. It was observed that cells adhered to the surface of the PDA-modified microspheres (PDPMs) from 1d of co-culture, and the number of cells continued to increase, resulting in the microspheres almost covered with stretched cells at 7d. In contrast, minimal cells were observed on the PDA-free microspheres (PMs) at 1d, and the unstretched shape of cells indicated that cell adhesion on the microspheres was unstable. Subsequently, the cells completely disappeared at 3d-7d, showing the effective antifouling property of microspheres. In conclusion, PCL porous microspheres proposed here showed switchable performances between antifouling and cells adhesion, which show potential for a wide range of applications.

### 3.7. In Vivo Histocompatibility and Degradation

**Figure 9:**
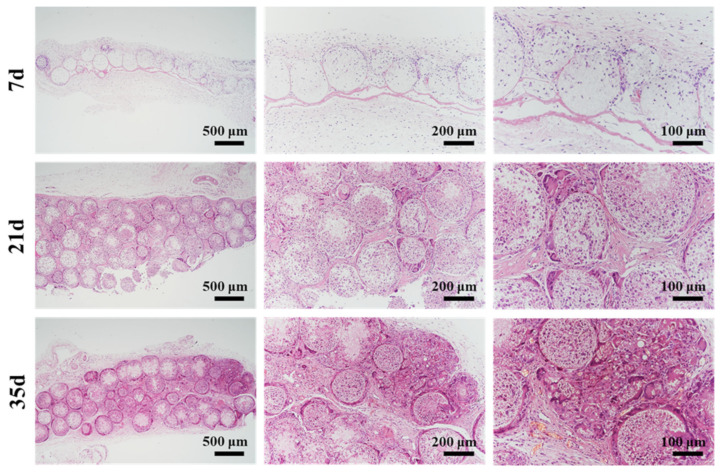
The results of H&E staining of implanted PDA-modified PCL porous microspheres after 7, 21, and 35 days.

The implanted microspheres were harvested after 7, 21, and 35 days for paraffin-embedded sectioning and H&E staining, and the results are shown in Figure 9. After seven days, it was observed that partial degradation in microspheres happened at the border, and some cells migrated into the microspheres. The PCL porous microspheres in vivo underwent much faster degradation than the solid PCL implants [41], which could be attributed to the highly porous structure. Additionally, abundant blood vessels and collagen fibers were observed between the microspheres, suggesting that the microspheres were well integrated with the surrounding tissue. A few lymphocytes were observed, which indicated a mild local inflammation. After 21 days, there was more degradation of the microspheres and tissue ingrowth. Some microspheres were observed to degrade into fragments. Finally, microspheres were almost completely degraded and replaced by the tissue after 35 days. In conclusion, PCL porous microspheres with PDA modification exhibited good histocompatibility and rapid degradation in vivo.

## 4. Conclusions

In summary, this study demonstrated an effective method combining a stable iso-density emulsion template with microfluidics for the controllable preparation of porous microspheres. This innovative iso-density emulsion was proven to remain stable for hours without introducing additional stabilizers, and the derived porous microspheres exhibited good uniformity in microsphere size and pore size distribution, which greatly facilitated the precise customization of microspheres. Using the iso-density emulsion template combined with microfluidics, PCL porous microspheres were prepared with the addition of amphoteric polymer PECL. Afterwards, the cytotoxicity test revealed that cells cocultured with PCL porous microspheres containing PECL could maintain a good proliferation trend. In the cell adhesion test, PCL porous microspheres exhibited anti-cell adhesion properties, while the simple PDA modification provided microspheres with good cell adhesion ability. Finally, PCL porous microspheres implanted subcutaneously showed rapid in vivo degradation and ingrowth of tissue and vessels into the porous structure of microspheres. Overall, this study provided an effective method for the precise fabrication of porous microspheres, and the obtained PCL porous microspheres exhibited potential biomedical applications in drug delivery, 3D cell culture, cell microcarrier construction, and in vivo injectable micro-scaffolds.

## Figures and Tables

**Table 1:** The preparation parameters of different microspheres. C_PECL_ (PECL concentration, *w*/*w*), C_PCL_ (PCL concentration, *w*/*w*), R_W/O_ (mass ratio of W to O), and T_H_ (homogenization time).

Microspheres	C_PECL_ (%)	C_PCL_ (%)	R_W/O_	T_H_ (%)
a	0	2.5	1/2.4	24
b	1			
c	0.5	2	1/2.4	24
d		3.5		
e	0.5	2.5	1/3	24
f			1/1.8	
g	0.5	2.5	1/2.4	12
h				48

## Data Availability

Not applicable.
